# Exploring the value of early laboratory indicators combined with pancreatitis activity scoring system in assessing the severity and prognosis of acute pancreatitis

**DOI:** 10.12669/pjms.39.5.7543

**Published:** 2023

**Authors:** Fang Xu, Xin Hu, Shu-ling Li

**Affiliations:** 1 Fang Xu Department of ICU, Affiliated Hospital of Hebei University, Baoding, Hebei, 071000, P. R. China.; 2 Xin Hu Electrocardiogram Room, Affiliated Hospital of Hebei University, Baoding, Hebei, 071000, P. R. China.; 3 Shu-ling Li Department of Critical Care Medicine, Baoding Lianchi District, People’s Hospital, Baoding, Hebei, 071000, P. R. China.

**Keywords:** Acute pancreatitis, Early laboratory examination, Severity, PASS score

## Abstract

**Objective:**

To investigate the value of early laboratory indicators combined with the pancreatitis activity scoring system in assessing the severity and prognosis of acute pancreatitis (AP).

**Methods:**

This is a retrospective study. A total of 160 patients with AP admitted to the Affiliated Hospital of Hebei University from February 2021 to February 2023 were enrolled and classified into three categories: mild acute pancreatitis (MAP), moderate severe acute pancreatitis (MSAP), and severe acute pancreatitis (SAP), with 80 cases with MAP and MSAP as the control group and 80 cases with SAP as the experimental group. The differences of inflammatory markers, blood routine, biochemical markers, coagulation markers and PASS score within 24 hours after admission were compared between the two groups, and independent risk factors for predicting AP severity were analyzed. Moreover, the diagnostic efficacy and prognostic value of independent risk factors were evaluated.

**Results:**

The PASS score as well as CRP, PCT, IL-6, WBC, N, AST, DD and PT were higher in the experimental group than in the control group. Logistic regression analysis suggested that PASS, IL-6, PCT and WBC were independent risk factors for predicting severity of AP. In addition, PASS had the highest diagnostic efficacy.

**Conclusion:**

Early elevation of PASS, IL-6, PCT and WBC in patients suffering from AP is of great significance in predicting SAP. PASS score combined with IL-6, PCT and WBC has important value in evaluating the severity and prognosis of AP.

## INTRODUCTION

Acute pancreatitis (AP) is an inflammatory reaction caused by the activation of pancreatic enzymes in the pancreas due to various etiologies, which leads to self-digestion, edema, hemorrhage and even necrosis of the pancreatic tissue.^1^,^2^ Eighty to 85% of AP patients have mild acute pancreatitis (MAP), but about 20% will develop moderate severe acute pancreatitis (MSAP) or even severe acute pancreatitis (SAP), with a morbidity and mortality rate of up to 13-35%.^3^ In terms of its prognosis, AP has a close bearing on the severity of the disease. To reduce the morbidity and mortality of AP and improve its prognosis, priority should be given to early and accurate determination of the severity of AP patients, and active interventions and treatment measures should be taken_._ Nowadays, many scoring systems are optional in the early assessment of the severity of AP patients, such as Ranson, G1asgow, BISAP score.^4^ Despite their value in assessing AP disease, they are not widely used in clinical practice due to various limitations.^5^

The Pancreatitis activity scoring system (PASS), a new pancreatitis activity scoring method developed jointly by several pancreatitis experts in recent years, has been shown to be valuable for the assessment of moderate to severe acute pancreatitis in preliminary studies.^6^ In view of this, routine laboratory indicators within 24 hours in patients with AP were retrospectively analyzed in this study, and these indicators were combined with the PASS score indicator to collectively assess AP condition. By doing so, we aimed to predict SAP earlier, comprehensively and accurately, and guide clinical early identification and intervention of SAP, thus ameliorating its prognosis.

## METHODS

This is a retrospective study. A total of 160 patients with Acute Pancreatitis (AP) admitted to the Affiliated Hospital of Hebei University from February 2021 to February 2023 were recruited as subjects. Patient data including demographic data, diagnosis of Acute Pancreatitis, were retrieved from electronic medical record systems. AP was classified into three categories according to the 2012 edition of the Revised Atlanta Classification of Acute Pancreatitis^7^, mild acute pancreatitis (MAP), moderate severe acute pancreatitis (MSAP), and severe acute pancreatitis (SAP), with 80 cases with MAP and MSAP as the control group and 80 cases with SAP as the experimental group. No significant difference was observed in the comparison of general data between the two groups, which was comparable (Table-I).

**Table-I:** Comparative analysis of general information of the two groups (*χ̅*±*S*) n=80.

Indicator	Experimental group	Control group	t	p
Male (cases %)	55	53	0.114	0.736
Age (years old)	54.96 ± 12.18	53.86 ± 11.65	0.584	0.560
Hypertension (cases %)	21	17	0.552	0.457
Diabetes mellitus (cases %)	16	13	0.379	0.538
BMI (kg/m^2^)	23.62 ± 2.53	23.37 ± 2.55	0.619	0.537
***Etiology***				
Biliary origin	52	60	1.905	0.168
Alcoholic	21	17	0.552	0.457
Other	7	3	1.707	0.191

p> 0.05.

### Ethical Approval:

The study was approved by the Institutional Ethics Committee of Affiliated Hospital of Hebei University on January 20, 2023 (No.: HDFYLL-KY-2023-009), and written informed consent was obtained from all participants.

### Inclusion criteria:

- Patients who met the diagnostic criteria for acute pancreatitis.^8^
- Patients with hospitalization time >48 hours.
- Patients aged 18 to 70 years.
- Patients with complete clinical data.
- Patients who themselves and their families were informed about this study and signed the informed consent form.

### Exclusion criteria:

- Patients with recurrent pancreatitis, pancreatic tumors, or pre-existing chronic pancreatitis.
- Patients with onset >72 hours at the time of admission.
- Patients with concurrent heart, lung, liver, or kidney diseases, or malignant tumors.
- Patients with incomplete clinical history information.
- Patients with underlying diseases such as definite infection, tumor or chronic liver and kidney disease at the time of admission.
- Patients with abnormal immune function or chronic inflammatory diseases.
- Patients who have recently taken drugs such as immunosuppressants and hormones that affect the outcome of the study.
- Patients with pre-existing hematologic disorders or recent oral medications affecting coagulation.
- Patients with mental illness or other reasons who could not cooperate to complete the study.

### Observation indicators and criteria:

Laboratory examination indicators, including inflammatory factor indicators such as C-reactive protein (CRP), calcitoninogen (PCT), interleukin 6 (IL-6); routine blood indicators such as white blood cell count (WBC), neutrophil count (N), lymphocyte count (L), platelet count (PLT); biochemical indicators such as total bilirubin (TB), direct bilirubin (DBIL), indirect bilirubin (IBIL), alanine aminotransferase (ALT), aspartate aminotransferase (AsT), adenosine deaminase (ADA), lactate dehydrogenase (LDH), triacylglycerol (TG), cholesterol (TC), high-density lipoprotein cholesterol (HDL), low-density lipoprotein cholesterol (LDL), blood glucose (GLU); and coagulation indicators such as D-dimer (DD), fibrinogen (Fib), prothrombin time (PT), activated partial thromboplastin time (APTT).

### PASS criteria:

(1) Organ failure (one point for each system of respiratory, circulatory, and renal, and cumulative for multiple system failures) × 100. (2) Inability to tolerate solid food (one point for intolerance, 0 point for tolerance) × 40. (3) SIRS (one point for each abnormal diagnostic criterion, cumulative for multiple abnormalities) × 25. (4) Abdominal pain (0-10 points on the pain rating scale) × 5. (5) Use of pain medication by intravenous route (converted to 1mg of morphine equivalent) × 5. Multiply the score for each of the above items by the weight number following the item and add the five scores to get the PASS score. The higher the score, the more severe the disease. A PASS score of >140 indicates moderate severe acute pancreatitis (MSAP), and vice versa for mild acute pancreatitis (MAP).^9^

### Statistical Analysis:

All data in this study were statistically processed using SPSS 20.0 software. Measurement data were expressed as (*X̅*±*S*), count data were compared as absolute value or component ratio, t test was employed for comparison between groups, and χ^2^ test was used for comparison of rates. A multi-factorial logistic regression model was used to analyze and find independent risk factors for predicting AP severity. Moreover, the area under the curve (AUC) and the optimal diagnostic threshold were used to calculate the optimal threshold, area under the curve (Auc), etc. for predicting SAP for each indicator. A 95% confidence interval was used. Pearson correlation was used for correlation analysis, with P<0.05 indicating a statistically significant difference.

## RESULTS

The PASS score as well as CRP, PCT, IL-6, WBC, N, AST, DD and PT were significantly higher in the experimental group than in the control group (P= 0.000) (Table-II). However, the remaining indicators were not significantly different between the two groups (p> 0.05).

**Table-II:** Comparative analysis of the differences in PASS score and laboratory examination indicators between the two groups (*χ̅*±*S*) n = 80.

Indicator	Experimental group	Control group	t	p
PASS score	147.63±13.52	122.94±10.96	12.686	0.000
CRP (mg/L)	27.49±5.41	23.63±6.24	4.176	0.000
IL-6 (ng/ml)	12.18±3.46	8.48±2.35	7.911	0.000
PCT (ng/ml)	0.83±0.21	0.35±0.18	15.448	0.000
WBC (10^9^/L)	11.58±3.72	8.29±1.96	6.986	0.000
N (10^9^/L)	9.73±4.17	6.06±3.76	5.845	0.000
L (10^9^/L)	1.24±0.24	1.21±0.25	0.863	0.390
PLT (10^9^/L)	197.18±7.12	195.25±5.35	1.929	0.056
TB (umol/L)	21.25±5.83	20.87±5.84	0.411	0.682
DBIL (umol/L)	6.71±1.71	6.37±1.69	1.290	0.199
IBIL (umol/L)	12.06±4.73	11.81±4.54	0.354	0.724
ALT (U/L)	26.44±5.72	25.87±5.56	0.640	0.523
AST (U/L)	26.91±5.50	19.84±5.67	8.007	0.000
ADA (U/L)	9.32±4.76	9.02±4.76	0.397	0.692
LDH (U/L)	243.51±8.57	243.42±2.68	0.091	0.928
TG (mmol/L)	2.38±0.34	2.28±0.33	1.848	0.066
TC (mmol/L)	4.73±2.34	4.64±2.18	0.262	0.794
HDL (mmol/L)	1.25±0.34	1.21±0.34	0.744	0.458
LDL (mmol/L)	2.48±0.35	2.43±0.36	0.887	0.376
GLU (mmol/L)	9.75±4.71	9.42±4.28	0.466	0.642
DD (ug/L)	1280.54±92.12	783.81±35.50	45.001	0.000
Fib (g/L)	4.43±2.35	4.28±2.10	0.440	0.661
PT (s)	14.37±2.51	10.41±2.66	9.679	0.000
APTT (s)	31.46±2.57	30.73±2.66	1.763	0.080

A multi-factorial logistic regression analysis was performed with PASS score and individual variables such as CRP, PCT, IL-6, WBC, N, AST, DD, and PT as independent variables and severity of patients’ disease as dependent variable. As shown in the results, PASS (p= 0.001), IL-6 (p= 0.000), PCT (p= 0.000) and WBC (p= 0.019) were independent risk factors for predicting AP severity (Table-III).

**Table-III:** Logistic regression analysis of independent risk factors for AP severity (*χ̅*±*S*) n=80.

Indicator	β value	SE value	Wald (^2^)	P value	95.0% CI
PASS*	0.152	0.045	11.502	0.001	0.064~0.240
CRP	0.064	0.068	0.900	0.343	0.069~0.197
IL-6*	14.960	3.440	18.915	0.000	8.218~21.701
PCT*	14.193	2.688	27.880	0.000	8.925~19.462
WBC*	0.280	0.120	5.477	0.019	0.046~0.515
N	0.189	0.097	3.774	0.052	0.002~0.379
AST	0.217	2883.355	0.000	1.000	-5651.054~5651.489
DD	0.190	59.561	0.000	0.997	-116.928~116.548
PT	2.667	1.062	0.000	0.999	-6741.195~6730.293

* P<0.05.

**Fig.1:**
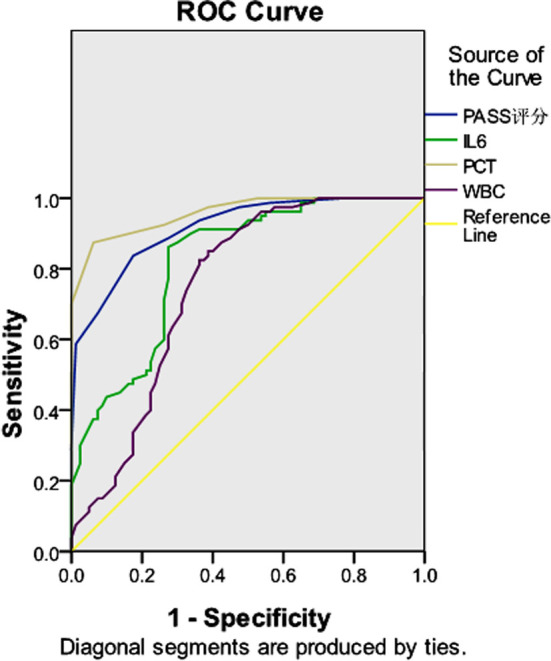
Diagnostic significance of PPASS score and independent risk factors in acute pancreatitis.

The predictive ROC curve of PASS score, IL-6, PCT and WBC in AP showed that the AUCs of PASS score, IL-6, PCT and WBC in predicting AP were 0.916, 0.819, 0.959 and 0.749, respectively, suggesting that PCT had the highest diagnostic efficacy, followed by PASS score and IL-6, while WBC had the lowest efficacy (Table-IV, Fig.1).

**Table-IV:** Diagnostic significance of PASS score and independent risk factors in AP (*χ̅*±*S*) n=80.

Indicator	Critical value	Sensitivity	Specificity	AUC	Youden index	95.0% CI	P value
PASS score	132.50	0.838	0.825	0.916	0.663	0.876~0.957	0.000
IL-6 (ng/ml)	10.75	0.863	0.725	0.819	0.588	0.755~0.884	0.000
PCT (ng/ml)	0.55	0.875	0.937	0.959	0.813	0.932~0.985	0.000
WBC (10^9^/L)	10.25	0.850	0.612	0.749	0.463	0.671~0.827	0.000

Correlation analysis suggested that PASS score, IL-6, PCT and WBC levels all increased with increasing AP severity, showing a positive correlation (Table-V). This indicates that PASS score, IL-6, PCT and WBC levels have a synergistic effect in determining AP severity.

**Table-V:** Correlation analysis of AP with PASS score, IL-6, PCT and WBC levels.

	PASS score		IL-6 (ng/ml)		PCT (ng/ml)		WBC (10^9^/L)	

	r value	P value	r value	P value	r value	P value	r value	P value
AP	0.725	0.000	0.553	0.00	0.798	0.000	0.432	0.000

## DISCUSSION

It was confirmed in our study that CRP, PCT, IL-6, WBC, N, AST, DD, PT and other indicators in SAP patients were significantly higher than those in MAP and MSAP patients. Consistently, Tian et al.^10^ also reported that SAP patients had more severe inflammatory response, coagulation dysfunction, decreased hepatic synthesis and renal excretory function. Multi-factorial logistic regression analysis suggested that PASS, IL-6, PCT and WBC were independent risk factors for acute pancreatitis predicting severity of the disease. Similarly, Van et al.^11^ in their study revealed that the levels of inflammatory factors in patients were upwardly increased with the increase of inflammatory response and positively correlated with the severity of the disease. PCT levels were considered to be positively correlated with AP severity by Zhang et al^12^ and were found to have predictive value for complications of AP in a study by Maciel et al^13^ as well, which is broadly consistent with our study. Pancreatic blood circulation disorders are now recognized as an important link in the development of severe pancreatitis.

AP is a common clinical acute abdomen.^14^ To date, no definitive conclusion about the pathogenesis of AP has been reached, and there are four recognized mechanisms: pancreatic enzyme self-digestion, pancreatic microcirculatory disorders, excessive inflammatory response of the body, and intestinal flora displacement.^15^ There is a close relationship between pancreatic alveolar injury and early inflammatory response in the pancreas.^16^ Coagulation and anticoagulation indicators, which are used in the prognostic evaluation of patients with AP, can reflect pancreatic injury, AP severity and visceral venous thrombosis (SVT). For this reason, laboratory indicators such as routine blood, biochemical and coagulation indicators are subject to change in AP patients and may be related to the severity of the disease. For AP patients, 24 hours after admission is the prime period of treatment. If SAP can be recognized early based on early routine screening laboratory indicators, and intensive care and comprehensive treatment can be given as early as possible, AP patients can have a much improved prognosis and a lower mortality rate.

A person with acute severe pancreatitis suffers disruption of the normal balance of his coagulation system due to activation of inflammatory factors, systemic inflammatory response syndrome. Study by Maduzia et al^17^ revealed that microcirculatory disorders of the pancreas act as a key as well as an early event in pancreatic necrosis. A comparison of SAP patients and MAP patients also showed significant abnormalities in all indicators of coagulation factors in both types of patients, suggesting the crucial importance of coagulation factor testing in the diagnosis of early AP. It was also demonstrated in our study that PT was an independent risk factor for predicting AP severity, and the level of PT gradually increased with the increase of severity.

Though certain assessment value on AP severity, laboratory indicators are subject to various confounding factors. To this end, it is more meaningful to predict the severity of SAP and determine the prognosis by using a combination of multiple indicators. The PASS is a new acute pancreatitis scoring system developed in recent years for the prognosis of moderate and severe acute pancreatitis and the assessment of its severity.^18^ As confirmed in our study, the PASS score was significantly higher in SAP patients than in the control group, and multi-factorial logistic regression analysis showed that PASS score was an independent risk factor for predicting AP severity. PSAA had the highest diagnostic efficacy with an area under the curve of 87.6%. Moreover, the PASS score was synergistic with IL-6, PCT and WBC in predicting AP severity, which is generally consistent with the findings of Wu Q et al.^19^

### Limitations:

This is a single-center study with a limited sample size, and relevant findings need to be validated in a multicenter, large sample, and multifaceted manner. Also, data are collected retrospectively, which may be biased, and the relevant comparative conclusions need to be verified by comparison in prospective studies. In response to this, more samples will be included in future clinical work, and prospective research methods will be adopted to further explore the optimal early diagnostic strategies and means to improve the prognosis of AP.

## CONCLUSION

Early elevation of PASS, IL-6, PCT and WBC in patients suffering from AP is of great significance in predicting SAP. PASS score combined with IL-6, PCT and WBC has important value in evaluating the severity and prognosis of AP.

### Authors’ Contributions:

**FX:** Carried out the studies, data collection, drafted the manuscript, is responsible and accountable for the accuracy and integrity of the work.

**XH:** Performed the statistical analysis and participated in its design.

**SL:** Participated in acquisition, analysis, or interpretation of data and draft the manuscript.

All authors read and approved the final manuscript.
